# Growth data of outlying plantations allows benchmarking the tolerance to climate extremes and drought stress in the European larch

**DOI:** 10.3389/fpls.2024.1404347

**Published:** 2024-05-31

**Authors:** Jesús Julio Camarero, Antonio Gazol, Cristina Valeriano, Marta Vergarechea, Nicolás Cattaneo

**Affiliations:** ^1^ Instituto Pirenaico de Ecología (IPE-CSIC), Zaragoza, Spain; ^2^ Laboratory of Tree-Ring Research, University of Arizona, Tucson, AZ, United States; ^3^ Department of Forest Management, Division of Forest and Forest Resources, NIBIO (Norwegian Institute for Bioeconomy Research), Ås, Norway

**Keywords:** afforestation, climate variability, extreme wet events, *Larix decidua*, drought

## Abstract

**Introduction:**

Plantations located outside the species distribution area represent natural experiments to assess tree tolerance to climate variability. Climate change amplifies warming-related drought stress but also leads to more climate extremes.

**Methods:**

We studied plantations of the European larch (Larix decidua), a conifer native to central and eastern Europe, in northern Spain. We used climate, drought and tree-ring data from four larch plantations including wet (Valgañón, site V; Santurde, site S), intermediate (Ribavellosa, site R) and dry (Santa Marina, site M) sites. We aimed to benchmark the larch tolerance to climate and drought stress by analysing the relationships between radial growth increment (hereafter growth), climate data (temperature, precipitation, radiation) and a drought index.

**Results:**

Basal area increment (BAI) was the lowest in the driest site M (5.2 cm2 yr-1; period 1988–2022), followed by site R (7.5 cm2 yr-1), with the youngest and oldest and trees being planted in M (35 years) and R (150 years) sites. BAI peaked in the wettest sites (V; 10.4 cm2 yr-1; S, 10.8 cm2 yr-1). We detected a sharp BAI reduction (30% of the regional mean) in 2001 when springto-summer conditions were very dry. In the wettest V and S sites, larch growth positively responded to current March and June-July radiation, but negatively to March precipitation. In the R site, high April precipitation enhanced growth. In the driest M site, warm conditions in the late prior winter and current spring improved growth, but warm-sunny conditions in July and dry-sunny conditions in August reduced it. Larch growth positively responded to spring-summer wet conditions considering short (1-6 months) and long (9-24 months) time scales in dry (site M) and wet-intermediate (sites S and R) sites, respectively.

**Discussion:**

Larch growth is vulnerable to drought stress in dry slow-growing plantations, but also to extreme spring wet-cloudy events followed by dry-hot conditions in wet fast-growing plantations.

## 1 Introduction

In Europe, climate change is increasing the frequency and intensity of extreme climate events such as droughts and heatwaves, a process which is very likely continuing in the future (Seneviratne et al., 2021). The occurrence of droughts and heatwaves is a factor potentially triggering forest mortality (Allen et al., 2010; Hammond et al., 2022), which has caused considerable forest die-off events and increases in background mortality rates across Europe (Senf et al., 2018; Gazol and Camarero, 2022). In addition, wet-cool extreme conditions can also impair tree growth and vigour through soil waterlogging or cold spells (Camarero et al., 2015, 2020). Thus, we need a more holistic framework considering the interplay of diverse climate extremes including cold and heat waves in addition to drought stress. An adequate assessment of the impacts of such climate stressors on natural and planted forests has important implications for their preservation, but also for the provisioning of their ecosystem services (Vacek et al., 2023).

Climate extremes such as droughts constrain the growth and performance of tree species, particularly in the species xeric bioclimatic limits, where it is crucial to track drought impacts (Danek et al., 2017; Saulnier et al., 2019; Escobar-Sandoval et al., 2021). However, tree species are commonly planted outside of their native ranges (Jansen and Geburek, 2016). Provenance trials established in the past (e.g., Szymański and Wilczyński, 2021) and plantations located in extreme environments (e.g., Gazol et al., 2022) offer valuable information for tracking how tree species respond to stress in extreme environments outside their natural distribution, information which may guide species translocation in the future.

South-western European forests are exposed to recurrent drought and heat waves that negatively affect tree growth (Vacek et al., 2023), but also to cold spells and spring frost events (Gazol et al., 2019; Camarero et al., 2020; Sangüesa-Barreda et al., 2021; Tonelli et al., 2022). Empirical evidence suggests that populations of tree species closer to their southern distribution range tend to be more vulnerable to drought stress (Camarero et al., 2021), although microsite conditions may modify this general pattern (Vilà-Cabrera and Jump, 2019). Overall, latitudinal gradients are valuable natural experiments to test for the response of plant performance to changes in limiting climate factors such as temperature and water availability as long as these factors vary in a straight linear fashion with latitude (De Frenne et al., 2013). In the case of planted, or non-native tree species, plantations along environmental gradients are useful to determine which climatic variables limit the establishment of the species (Pötzelsberger et al., 2020). Moreover, dendrochronological techniques can be employed to decipher which climate conditions limit growth in these marginal plantations (Zas et al., 2020; Gazol et al., 2022).

Among conifers, species distribution models indicate that southern populations of deciduous larch (*Larix*) species growing in xeric sites would show greater recession and thinning related to warming-related drought stress (Mamet et al., 2019). Indeed, growth decline and retraction of larch forests have been documented in xeric mountain areas from Asia (Dulamsuren et al., 2010; Xu et al., 2017; Zhou et al., 2021). Growth models using as input different climate scenarios also suggest a decline in productivity of mountain larch (*Larix principis-rupprechtii*) populations in north China linked to climate warming (Duan et al., 2022; Cheng et al., 2023). However, such information is lacking for southern Europe where larch species, including the European larch (*Larix decidua* Mill.), have been planted in mountain areas (Da Ronch et al., 2016).

The European larch is a deciduous and light-demanding tree species mainly found in the mountains ranges of central and Eastern Europe under dry and continental conditions (Da Ronch et al., 2016). The species grows naturally along altitudinal gradients in the Alps and the Carpathian Mountains where it can extend from montane and subalpine forests to the treeline (Danek et al., 2017; Saulnier et al., 2019). In addition to timber provision and biodiversity conservation, natural larch forests protect from several hazards (e.g., avalanches, landslides) in mountain areas (Hillebrand et al., 2023). The capacity of this pioneer conifer to colonize post-agricultural lands (Zeidler et al., 2022), its fast growth and the value of its wood for timber and wood construction (Da Ronch et al., 2016) make it a valuable commercial tree species. Thus, this species has been widely planted in Europe for several centuries translocating genes between populations and sites (Jansen and Geburek, 2016).

The growth of European larch responds to climate variability in different regions. For instance, its growth is sensitive to the thermal conditions during spring in the Carpathians, probably because this is the beginning of the cambial activity (Danek et al., 2017; Danek and Danek, 2022). At high elevation, air temperature is the main factor limiting the growth of the species in the Alps, and warmer conditions linked to climate change are relaxing growth limitations (Obojes et al., 2022). However, at low elevation the growth of European larch is limited by high summer temperatures and dry spring conditions suggesting its vulnerability to climate warming and future aridification (Danek et al., 2017; Saulnier et al., 2019). Interestingly, Izworska et al. (2022) showed that summer precipitation was the main factor limiting larch after windthrow storms. Szymański and Wilczyński (2021) found that the response of larch growth to climate varied between provenances. These studies were mostly carried out in wet-cool sites or in sites with wet summers. However, it remains unknown what will be the capacity of the European larch to thrive in Mediterranean continental sites subjected to summer dry conditions. We argue that obtaining information on growth responses to climate and drought severity in these southern European larch plantations would be useful to better benchmark the climatic tolerance of the species.

In this study, we used dendrochronological methods to reconstruct the radial growth patterns of European larch in four plantations subjected to Mediterranean climate conditions in northern Spain. Despite precipitation is not an important factor limiting the growth of the species in most sites across central and eastern Europe (e.g., Rozenberg et al., 2020), excepting in low-elevation Alpine dry sites (Lévesque et al., 2013, 2014; Obojes et al., 2018), we expect that water scarcity during summer would reduce larch growth, particularly in the driest study site. Due to the climatic marginality of some of the study plantations, located near the species bioclimatic limits of drought tolerance, we expect that tree growth will be limited by warm-hot droughts.

## 2 Material and methods

### 2.1 Study sites

**Figure 1:**
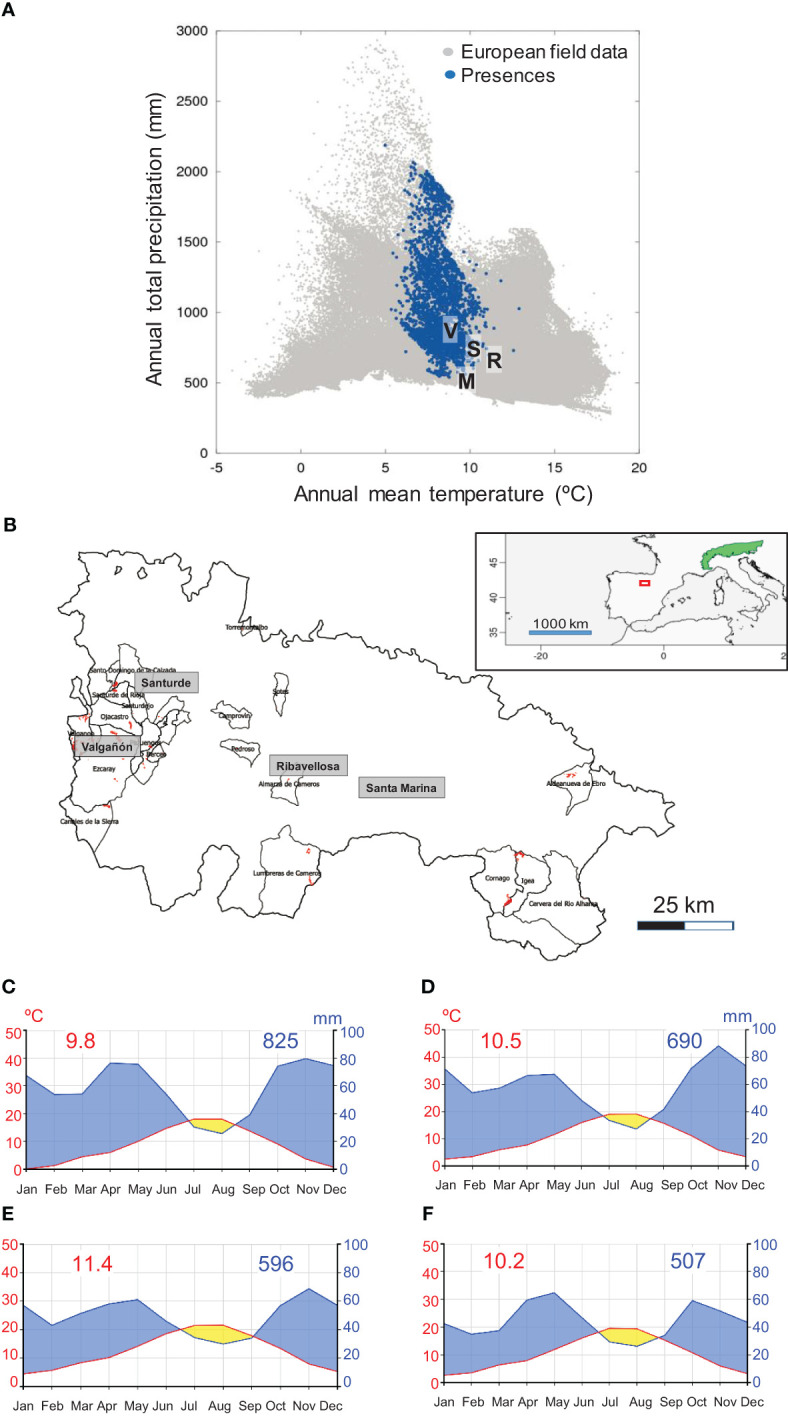
**(A)** Situation of the four study sites (V, S, R, and M) in the larch bioclimatic space. The blue symbols show locations of larch and grey symbols indicate European field data. Modified from De Rigo et al. (2016). **(B)** Natural range (green patch) of the alpine European larch subspecies (*Larix decidua* subsp. *decidua*) and location of the study area (red box). Location of the four study sites in La Rioja, northern Spain (red patches show larch plantations and polygons correspond to municipalities). Climate diagrams of the study sites: **(C)** Valgañón, **(D)** Santurde, **(E)** Ribavellosa and **(F)** Santa Marina. Red and blue numbers show mean annual temperatures and total annual precipitations, respectively. Climate diagrams were drawn using the ClimateCharts.net webpage (Zepner et al., 2020).

In south-western Europe, European larch (hereafter larch) plantations are mainly restricted to those areas with a marked Atlantic influence that favour the growth of the species by buffering it against the Mediterranean summer drought. This is the case of the four study sites located in La Rioja autonomous community, northern Spain (**Figure 1**). The four sites are located far from the core species distribution area (Jansen and Geburek, 2016). In this region, the NW-SE alignment of the major mountains (Iberian System range) leads to drier conditions south-eastwards as the relevance of rainfall from Atlantic fronts diminishes and that of Mediterranean-continental conditions increases (dry summers, lower winter-spring precipitation and higher inter-annual rainfall variability, greater thermal amplitude). Therefore, the north westernmost Valgañón site is the wettest, whereas the south eastern Santa Marina site is the driest (**Table 1**).

**Table 1:** Characteristics of the larch plantations sampled in La Rioja, northern Spain.

Site (code)	Latitude °N	Longitude °W	Elevation (m a.s.l.)	Slope (%)	No. sampled trees	Dbh (cm)	Tree height, range (m)	Other planted tree species
Santurde (S)	42.4044	3.0022	1056	32	15	35.7 ± 1.3c	15−18	*Pinus sylvestris*
Valgañón (V)	42.3031	3.0906	1127	37	16	29.2 ± 0.8b	18−20	*Pinus sylvestris*
Ribavellosa (R)	42.2396	2.5905	1072	40	20	40.3 ± 3.0c	15−20	*Picea abies*, *Pinus sylvestris*
Santa Marina (M)	42.2240	2.3706	1190	15	20	20.3 ± 0.5a	10−12	*Pinus nigra*, *Pinus sylvestris*

Dbh is the diameter at breast height. Different letters indicate significant (*p* < 0.05) differences among sites according to Mann-Whitney tests.

The aspect of sampled sites was W-NW and the slope ranges 15−40%. Soils are acid (Valgañón, Santurde and Santa Marina) or basic (Ribavellosa), deep and they are developed on shales (Valgañón, Santurde), quartz sandites (Santa Marina) or limestone (Ribavellosa) substrates. Soil depth was not measured in the field but soil depth seems to be the lowest in Santa Marina where soils are also very rocky. The planting distance between trees varied from 3 to 5 m and trees were planted along terraces separated by 5−7 m. Trees were neither recently thinned nor pruned excepting in Santurde, where a light thinning was done in 2008. The natural forest vegetation corresponds to oaks (*Quercus petraea*, *Quercus pyrenaica*, *Quercus ilex*, *Quercus faginea*) and European beech (*Fagus sylvatica*). Understory vegetation is dominated by ferns (*Pteridium aquilinum*) and heather (*Erica arborea*) in the wet Valgañón and Santurde sites. In the driest site (Santa Marina), the understory was dominated by laurel-leaf rock rose (*Cistus laurifolius*), whereas in Ribavellosas the understory was dominated by ferns and grasses. The origin of the seeds used for plantation is unknown, but probably corresponds to central European forests (cf. Jansen and Geburek, 2016).

The four sites are characterized by a Mediterranean climate with summer drought and precipitation maxima in winter-spring and autumn (**Figure 1**). Summer precipitation ranges 140−170 mm and there is a period of summer drought in all sites, but it is shorter in Valgañón and Santurde (July) than in Ribavellosa and Santa Marina (July−August; **Figure 1**).

### 2.2 Field sampling and tree-ring data

In each site, we sampled between 15 and 20 dominant individuals, measured their diameter at breast height (DBH), and estimated their maximum height. In each individual, we extracted two increment cores at breast height (1.3 m) using a Pressler increment borer (Haglöf Sweden AB) and cored perpendicular to the maximum slope.

The collected wood samples were processed using standard dendrochronological methods (Fritts, 1976). Cores were air dried, mounted in wooden supports, and sanded with sandpapers of different grains until growth rings were visible. Then, these samples were scanned at a resolution of 2,400 dpi using an Epson Expression 10,000-XL scanner (Epson, Suwa, Japan). After that, samples were visually cross-dated, and ring widths were measured to the nearest 0.001 mm from the pith to the bark considering two radii for each sampled tree. The CooRecorder and CDendro software (Larsson and Larsson, 2022) were used to cross-date and measure the samples. The quality of the visual cross-dating was assessed with the COFECHA software (Holmes, 1983). This process is based on the computation of correlations between a site mean chronology and the ring-width series of each sampled tree.

### 2.3 Processing tree-ring data

To have an accurate indicator of biomass increment, we transformed ring-width measures into basal area increment (BAI; Biondi and Qeadan, 2008). Ring-width measurements from two increment cores were averaged within individuals and the within each sites. The BAI was calculated outside-in assuming concentric growth and using the formula:


(1) $$BAI\;=\;\pi \;\left(r_{t}{\;}^{2}-r_{t-1}{\;}^{2}\right)$$

where *r_t_* and *r_t-1_* represent the width of the rings in the year *t* and *t*-1, respectively (Equation 1). Finally, mean BAI series were calculated in each site by averaging the BAI series of the individuals sampled in each site.

To calculate climate-growth relationships, the individual ring-width series were converted into series of ring-width indices through standardization and detrending (Fritts, 1976). This allows removing size-related trends in ring-width data and emphasize high-frequency growth variability. We fitted 33-year cubic smoothing splines with a 50% frequency response cut-off to individual ring-width series and obtained ring-width indices (RWI) by dividing observed by fitted values. The length of the spline was selected to mainly retain annual to decadal growth variability. Then, we fitted autoregressive models to remove most of the first-order autocorrelation in series of dimensionless ring-width indices. The obtained residual or pre-whitened individual series were averaged using a bi-weight robust mean to obtain mean residual series for each site (Fritts, 1976).

Lastly, we calculated several dendrochronological statistics for the common, best-replicated period (1988–2022). This period was defined based on the values of the Expressed Population Signal (EPS). We considered the period with EPS ≥ 0.85 as the best-replicated one, during which the calculated chronologies approached the theoretically perfect chronologies (Wigley et al., 1984). We also characterized the mean site chronologies by calculating: the mean ring-width values, the mean first-order autocorrelation of ring widths (AR1), which accounts for year-to-year persistence in growth, the mean sensitivity (MS), which reflects relative changes in ring width between consecutive rings, the mean interseries correlation (rbar), and the EPS (Briffa and Jones, 1990). The MS and rbar were calculated on detrended series of RWIs. To summarize the relationships among site series of growth indices and to highlight pointer years, we calculated a Principal Component Analysis (PCA) on the variance-covariance matrix of residual chronologies considering the best-replicated period (1988–2022). We kept the two first principal components (PC1 and PC2) because they accounted for most of the 75% of the variability. A PCA biplot showing the sites’ loadings and the years’ scores was drawn to assess the similarity among sites’ growth series and years.

The dplR package was used to process dendrochronological data including the calculation of statistics (Bunn, 2008, 2010; Bunn et al., 2023).

### 2.4 Climate and drought severity data

Due to the lack of long-term and homogeneous local climate data near the sampled sites, we downloaded monthly climate data (mean maximum and minimum temperatures, total precipitation, mean radiation) from the 0.25°-gridded E-OBS ver. 27.0e dataset for the period 1970–2022 (Cornes et al., 2018). Temperature, precipitation and radiation data were also detrended using 33-year long splines via subtraction to enhance the climate signal of RWI series (Ols et al., 2023). Additionally, 0.5°-gridded monthly SPEI data from the study area were collected to assess SPEI changes and related growth responses at a regional scale considering the area delimited by coordinates 2.0−3.0°W and 42.0−42.5°N. The SPEI has positive and negative values for wet and dry conditions, respectively, and it is based on a cumulative climatic water balance calculated as differences between precipitation and potential evapotranspiration (Vicente-Serrano et al., 2010). Climate and SPEI data were obtained from the Climate Explorer (https://climexp.knmi.nl/) and the global (https://spei.csic.es/map/maps.html) and Spanish SPEI (http://monitordesequia.csic.es/) webpages, respectively.

In order to characterize the climate conditions in 2001, a year marked by minimal growth across all study sites, we conducted an extensive search for climate stations with long-term homogenous climate series in the study region using the R package *climaemet* (Pizarro et al., 2021). The only climate station present in the region was the one corresponding to the Logroño airport (42.4650, 2.4456 W, 384 m), and we downloaded daily climate data (mean, maximum and minimum temperature, total precipitation, and minimum and maximum atmospheric pressure; period 1951−2022).

### 2.5 Statistical analyses

Trends of annual climate variables and log-transformed BAI (period 1988–2022) were assessed using non-parametric Kendall tau (τ) tests. Ring-width data were compared among sites using Mann-Whitney tests. We used the abrupt growth change method (Schweingruber et al., 1990) to search for the presence of positive and negative pointer years (extremely wide and narrow rings, respectively). This method compares the growth observed in a particular year with the growth observed in the preceding *n* years. This method for pointer year detection was selected because it captures abrupt growth changes, although other methods exist and have their pros and cons (Jetschke et al., 2019). We compared the standardized individual series of ring-width indices in each year with the average value in the preceding three years. We used default settings and considered negative pointer years when 75% of the individuals in a site presented growth reductions higher than 40%, and positive pointer year when 75% of the individuals presented growth enhancements higher than 60%.

The relationships between growth variability and the SPEI drought index at different temporal scales were calculated for the 1988–2022 period by using regularly bootstrapped Pearson correlation coefficients. In particular, we correlated the residual site chronologies of each site and also the PC1 scores with the SPEI (drought index) at 1, 3, 6-, 9-, 12- and 24-month and 1-to-24-month scales, respectively. Correlations were obtained for each month of the year, from September of the previous year of growth (*t*-1) to September of the current year (*t*). Climate- and SPEI-growth correlations were also assessed at the regional level by using the PC1 and PC2 scores. In the case of SPEI-PC1 correlations, the temporal window of analyses went from January to December. We used 1000 iterations to establish the significance of each correlation coefficient.

All statistical analyses were performed using the R Studio platform and the R statistical software (R Development Core Team, 2023). The “Res.comp” function from PointRes package (van der Maaten-Theunissen et al., 2015, 2021) was used to calculate pointer years. The vegan package was used to calculate the PCA (Oksanen et al., 2024). Finally, we used the “dcc” function from the treeclim package (Zang and Biondi, 2015) to calculate the correlations between residual ring-width series and climate variables or SPEI.

## 3 Results

### 3.1 Climate trends

Annual mean maximum and minimum temperatures and the SPEI showed significant positive and negative trends, respectively, indicating progressively warmer and drier conditions (**Supplementary Figure S1**). We found a significant (*p* < 0.05) negative trend in the precipitation series of site R.

### 3.2 Radial increment data

**Figure 2:**
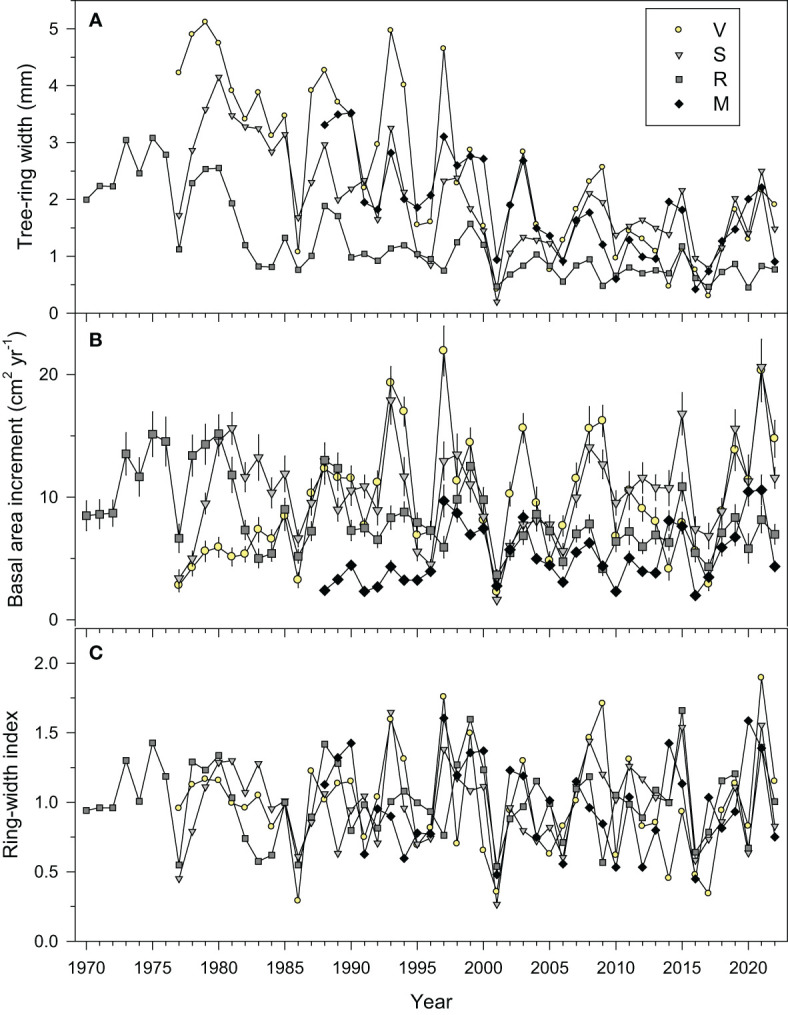
Mean series of **(A)** tree-ring widths, **(B)** basal area increment (means ± SE), and **(C)** ring-width indices measured in the four study sites (V, Valgañón; S, Santurde; R, Ribavellosa; M, Santa Marina). The displayed periods correspond to the best-replicated timespans.

Larch BAI (5.2 cm^2^ yr^-1^) was significantly lower in Santa Marina (hereafter site M) than in the rest of the sites (**Table 2**, **Figure 2**). Then, the lowest BAI (7.5 cm^2^ yr^-1^) was found in Ribavellosa (hereafter site R). The BAI did not significantly differ between the two wettest sites, Valgañón (hereafter site V; 10.4 cm^2^ yr^-1^) and Santurde (hereafter site S; 10.8 cm^2^ yr^-1^). Sites V and R showed significant positive and negative trends, respectively, of log-transformed BAI data (V, τ = 0.21, *p* = 0.042; R, τ = -0.23, *p* = 0.021).

**Table 2:** Tree-ring width statistics calculated for the for the common, best-replicated period (1988–2022).

Site (code)	Timespan	No. radii	Basal-area increment (cm^2^ yr^-1^)	AR1	MS	Rbar	EPS (best-replicated timespan)
Santurde (S)	1967−2023	30	10.8 ± 0.8c	0.69	0.51	0.54	0.89 (1977−2022)
Valgañón (V)	1972−2022	31	10.4 ± 0.7c	0.57	0.57	0.61	0.97 (1977−2022)
Ribavellosa (R)	1873−2022	34	7.5 ± 0.4b	0.72	0.43	0.58	0.95 (1951−2022)
Santa Marina (M)	1983−2022	35	5.2 ± 0.4a	0.53	0.50	0.56	0.90 (1988−2022)

Values are means ± SE. Different letters indicate significant (*p* < 0.05) differences among sites according to Mann-Whitney tests.

The oldest and youngest trees were sampled in R and M sites with ages at 1.3 m of 150 and 35 years, respectively. Considering all trees or only the trees from site R we detected negative relationships between age and mean tree-ring width (**Supplementary Figure S2**). However, this association was due to the existence of two age classes in site R with mean ages of 57 and 136 years, respectively.

We found a strong BAI reduction in 2001 when the mean growth rate of all sites (2.6 cm^2^ yr^-1^) was only 30% of the mean of the 1980−2022 period (8.5 cm^2^ yr^-1^). The relative BAI drop in 2001 was stronger in the wettest V (21%) and S (16%) sites than in the intermediate R (49%) and dry M (53%) sites.

The ring-width series showed a higher growth variability in V and S than in R and M (**Figure 2**), which was confirmed by the higher mean sensitivity of the indexed ring-width series of these two sites (**Table 2**). The highest first-order autocorrelation (0.72) was found in site R, whereas the highest rbar (0.61) and EPS (0.97) values were found in site V. All sites’ chronologies showed EPS values above the 0.85 threshold confirming a reliable cross-dating.

### 3.3 Relationships among site series of growth indices

**Figure 3:**
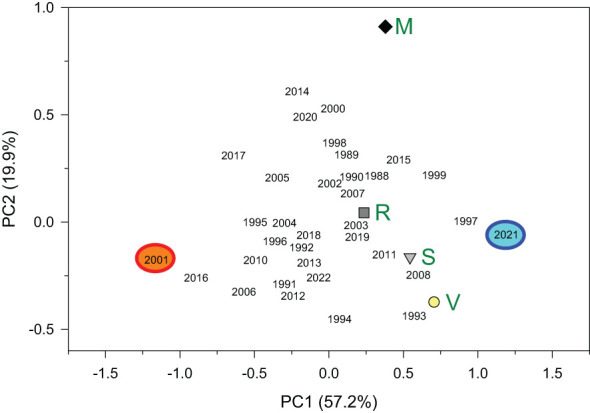
Biplot showing the four sites’ loadings (green letters; V, Valgañón; S, Santurde; R, Ribavellosa; M, Santa Marina) in the first (PC1) and second (PC2) principal components of a PCA. The years’ scores are also shown for the period 1980−2022 and the two extreme years are indicated (2001, narrow ring; 2021, wide ring). The percent of variance explained by each PC is shown between parentheses.

**Figure 4:**
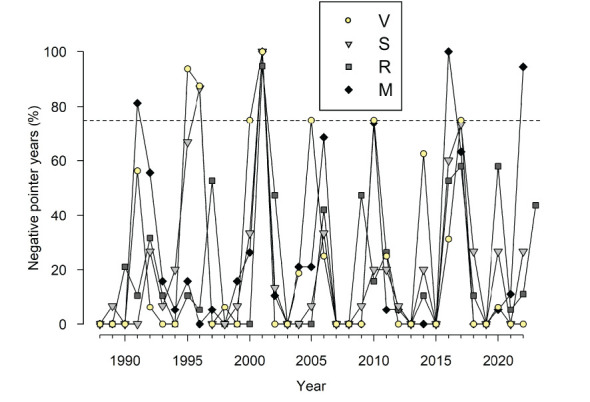
Percentage of trees showing negative pointer years in the four study sites during the period 1988−2022. The horizontal dashed line shows the 75% value above which a particular event can be considered a pointer year.

The PCA showed that the chronologies from the nearby V and S sites were more similar than the chronologies from R and V sites and R and M sites (**Figure 3**; **Supplementary Table S1**). Most growth variability was accounted for by the PC1 (57.2%) showing a growth maximum in 2021 and low growth indices in 2001. Abrupt growth reductions occurred in all sites, but the only year with extreme growth reductions common across sites was the year 2001 (**Figures 2**, **4**). Other years with strong growth reductions in different sites were 1995−1996 (S and V sites) and 2016 (M site).

### 3.4 Growth responses to climate and drought at regional and local scales

**Figure 5:**
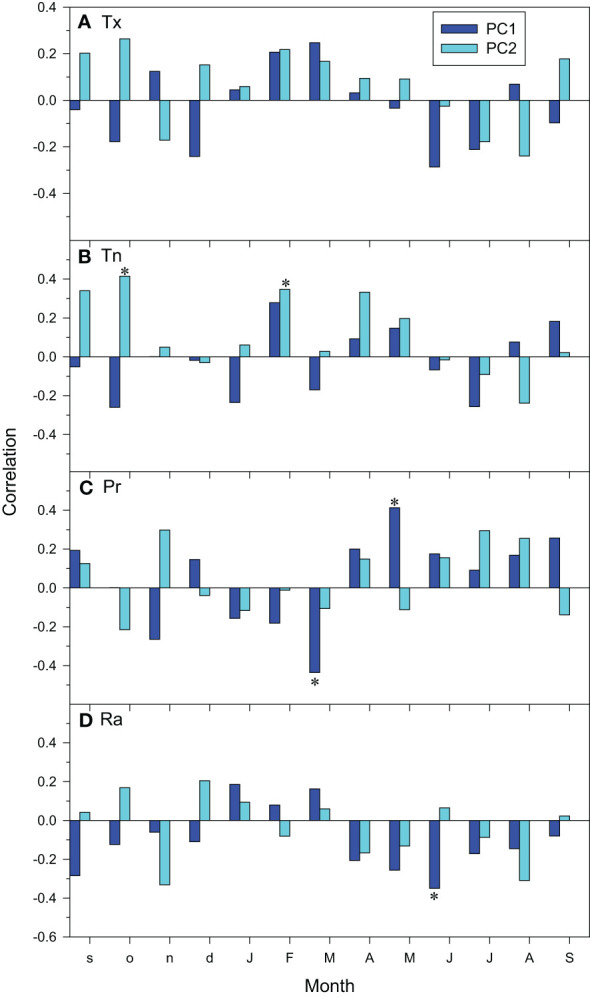
Regional climate-growth relationships assessed as correlations between monthly climate variables (**(A)**, Tx, mean maximum temperature; **(B)**, Tn, mean minimum temperature; **(C)**, Pr, precipitation; **(D)**, Ra, radiation) and series of the PC1 and PC2 scores. Months of the previous and current years are abbreviated by lowercase and uppercase letters, respectively. Asterisks indicate significant (*p* < 0.05) correlations (Pearson correlation coefficients).

At the regional scale, the climate-growth relationships showed that the PC1 increased as March precipitation and June radiation decreased and as May precipitation increased (**Figure 5**). The PC2 scores only showed significant relationships with minimum temperatures increasing as previous October and current February minimum temperatures did.

**Figure 6:**
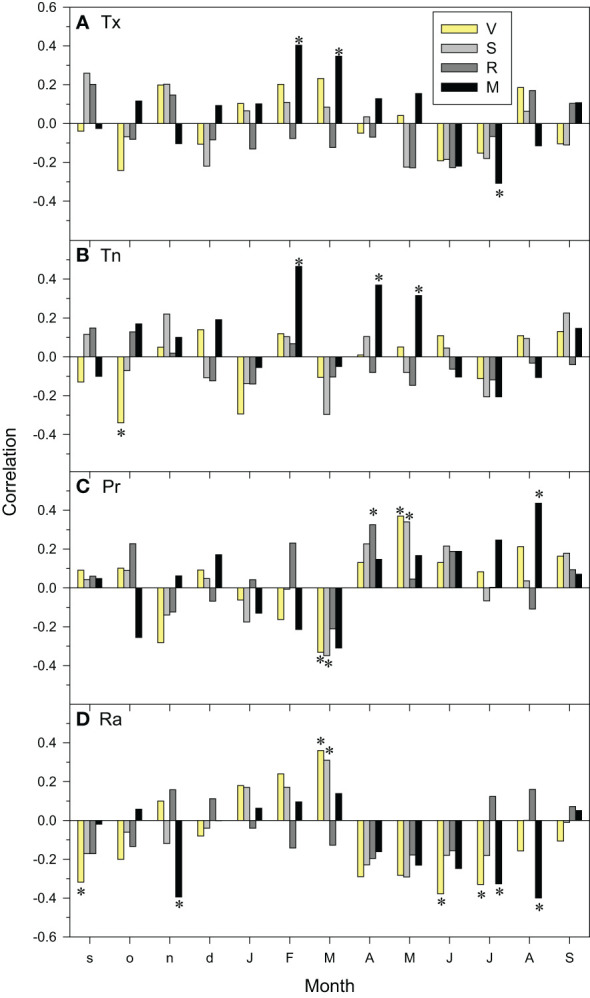
Climate-growth relationships assessed as correlations between monthly climate variables (**(A)**, Tx, mean maximum temperature; **(B)**, Tn, mean minimum temperature; **(C)**, Pr, precipitation; **(D)**, Ra, radiation) and site series of ring-width indices. The bars of different fills correspond to the four study sites (V, Valgañón; S, Santurde; R, Ribavellosa; M, Santa Marina). Months of the previous and current years are abbreviated by lowercase and uppercase letters, respectively. Asterisks indicate significant (*p* < 0.05) correlations (Pearson correlation coefficients).

At local scale, the M site was the most sensitive to temperature variability showing positive relationships between growth indices and the maximum temperatures in current February and March, and the minimum temperatures in February, April and May (**Figure 6**). In contrast, negative relationships were observed between current July maximum temperatures and growth indices, suggesting a vulnerability to heat-induced drought stress. This was confirmed by a positive correlation with August precipitation and negative correlations with July-August and prior November radiation levels. Elevated minimum temperatures in the previous October reduced growth in site V (**Figure 6**). Remarkably, high precipitation in current March reduced growth in V and S sites whilst low radiation levels enhanced it. In contrast, high precipitation in current May and April enhanced growth in V-S and R sites, respectively, whereas high radiation levels in the prior September and current June-July reduced growth in the V site.

**Figure 7:**
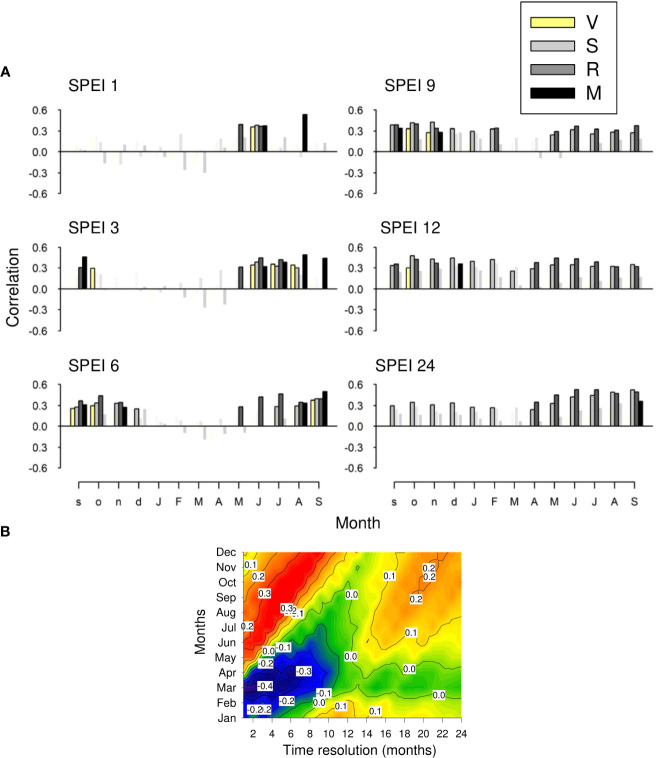
**(A)** Relationships (Pearson correlation coefficients) observed between monthly values of the regional SPEI drought index and site series of ring-width indices. Symbols in color indicate significant (*p* < 0.05) correlations, which were calculated for 1- (SPEI 1), 3- (SPEI 3), 6- (SPEI 6), 9- (SPEI 9), 12- (SPEI 12) and 24-month (SPEI 24) long scales. **(B)** As plot **(A)** but based on correlations with the PC1 scores and considering time resolutions from 1 to 24 months and a temporal window from January to December. Correlations with *r* > |0.34| are significant.

The correlation between growth variability and the SPEI calculated at different time scales during the summer and the previous autumn suggested the importance of the water balance at short scales (1-3 months), particularly in the driest M site (**Figure 7A**). In the S and R sites, the growth indices positively responded to spring-summer SPEI at longer scales (12 and 24 months). At a regional scale, SPEI-PC1 correlations showed that growth indices increased as spring (March, April) and summer-autumn (June to October) SPEIs decreased and increased, respectively (**Figure 7B**). In absolute terms, correlations of the regional SPEI with the PC1 scores peaked at short time scales (1 to 6 months).

### 3.5 Climatic conditions during 2001

We further explored the climatic conditions in 2001 when radial increment was minimum in the sampled larch plantations (**Figures 2**, **3** and **4**). In this year, there was a sharp drop in atmospheric pressure followed by cold conditions from late April to early May, and very dry conditions in June (**Supplementary Figures S3** and **S4**).

## 4 Discussion

This study explores the capacity of European larch to thrive in water-limited environments in southern European mountains, far from its natural distribution area. We sampled here four plantations that thrive in seasonally dry sites in which summer drought might negatively affect the growth of the species. The results do not fully confirm our hypotheses, since we found that European larch is less impacted by drought than expected. The negative impact of higher summer temperature, rising evapotranspiration rates, on growth and the positive influence of summer precipitation was only found in the driest M site, where short-term droughts also constrained growth in agreement with our last hypothesis. This indicates that summer drought was only important in that very marginal M site, where growth rates were the lowest. Indeed, several of these Spanish sites (V, S, and R) are not drier than some inner-Alpine dry valley sites where larch grows, but they are 3-4°C warmer on the yearly average and even warmer if only considering maximum temperatures in summer.

The PC1 scores accounted for most growth variability of the study larch plantations as indicated their correlations with the main climate drivers (spring precipitation and radiation). The sharpest decrease in radial growth observed in 2001 coincided with very dry June conditions reflecting the primary role played by late spring and early summer precipitation on larch wood production. This was supported by the positive correlation found between the June 3-month SPEI and all sites’ series of ring-width indices. Warm conditions in the prior late winter and current early spring improved growth in the driest M site, indicating a dependence on early-season growing conditions and probably inducing an earlier start of the xylem onset. In this xeric site, growth depended more on a positive climatic water balance in summer, explaining its positive correlation with short SPEI scales, whilst the coupling between growth and long droughts (9-12 months) in the wettest V and S sites explains their dependence on spring precipitation and a low water deficit in the prior autumn. In the old plantation from site R, growth was also enhanced by high April precipitation.

The linkages between poor growth in 2001 and dry spring-to-summer conditions are supported by phenological larch data. In larch species needles start emerging from March to late April and the onset of xylem formation occurs from mid to late May (Antonova and Stasova, 1997; Moser et al., 2010; Jiang et al., 2015; Wang et al., 2023). In the Swiss Alps, larch radial growth peaked from late May to mid-July (Moser et al., 2010). Therefore, it is reasonable to argue that the very dry and rather hot April to June conditions negatively affected larch earlywood formation and growth rate.

In several conifers from cold biomes, the growth peak occurred around the time of maximum day length in late June, suggesting that photoperiod could constrain cambial activity in the late growing season (Rossi et al., 2006). This does not agree with the negative relationship observed between June-July radiation and growth indices in the wettest V site. Therefore, the positive association between day length and growth rate observed in cold biomes does not seem to hold in warmer, subtropical, Mediterranean biomes.

In general, European larch plantations do not cope with dry conditions because they show a low recovery capacity from drought years (Eilmann and Rigling, 2012; Lévesque et al., 2014). The negative impacts of climate warming and more arid conditions on low-elevation populations of several larch species growing in xeric sites have been well documented in Eurasia (Dulamsuren et al., 2010; Zhang et al., 2018; Rozenberg et al., 2020; Danek and Danek, 2022; Ning et al., 2022).

Nevertheless, our study had several limitations. First, our climate-growth correlations were based on a very short time frame (35 years) and longer series would produce more robust estimates. Second, we did not fully consider potential effects on growth trends from site conditions, including factors such as stand basal area, tree-to-tree competition, tree species mixture or tree age. In the driest M site, understory was sparse so we expect little competition with trees for soil water. However, the deep soils in sites V, S and R with a good water storage capacity could buffer meteorological drought. Not only climate warming but land-use changes could impact the study plantations because planted larches coexisted with more competitive species such as Scots pine. For instance, in the Alps, the very light-demanding larch is being outcompeted by pine species at lower elevations, due to the cessation of historical land uses (e.g., logging), but it is expanding upwards above the treeline (Carrer et al., 2013). Species diversity and competition showed minor effects on the growth response of European larch to drought (Charlet de Sauvage et al., 2023). Unfortunately, no provenance information was available, but this is common with old plantations in Europe (Lévesque et al., 2013, 2014).

Long-term tree-ring data from old plantations (e.g., site R) and xeric sites (e.g., site M) allow assessing future risks of increasing aridification to decide where and when larch or other non-native tree species should be used for reforestation. According to recent surveys, there is a strong interest in planting non-native tree species in Spain for productive purposes (Pötzelsberger et al., 2020). Our findings show the high growth potential of European larch in wet-cool sites from northern Spain (e.g., V and S sites), but also illustrate the vulnerability of these plantations to extreme climate events including cool-wet episodes followed by warm-hot conditions in spring.

## 5 Conclusions

European larch trees planted far from the main species distribution area represent a suitable setting to assess growth responses to climate extremes of non-native tree species. Larch showed the lowest growth rates and was more negatively impacted by short-term droughts in the driest site. In contrast, the fastest growth rates were found in the wettest sites, where growth was constrained by long-term summer droughts and also by rainy and cloudy conditions in March. Indeed, a sharp growth drop was linked to low pressures triggering wet, cool and cloudy conditions in early spring followed by dry-hot conditions in early summer. Our findings show that growth drops in geographically and climatically marginal plantations may be triggered by different climate extremes. Planting trees in climatically marginal sites, far from the species distribution area, is risky if more climate extremes threaten some of these plantations.

## Data availability statement

The raw data supporting the conclusions of this article will be made available by the authors, without undue reservation.

## Author contributions

JC: Conceptualization, Data curation, Formal analysis, Funding acquisition, Investigation, Methodology, Project administration, Resources, Software, Supervision, Validation, Visualization, Writing – original draft, Writing – review & editing. AG: Conceptualization, Data curation, Funding acquisition, Investigation, Methodology, Software, Visualization, Writing – review & editing. CV: Data curation, Formal analysis, Investigation, Methodology, Resources, Software, Writing – review & editing. MV: Conceptualization, Investigation, Methodology, Writing – review & editing. NC: Conceptualization, Investigation, Methodology, Writing – review & editing.
