# Supplementary material for: Growth data of outlying plantations allows benchmarking the tolerance to climate extremes and drought stress in the European larch
Source: Front Plant Sci. 2024 May 31;15:1404347. doi: 10.3389/fpls.2024.1404347 (PMC11176551; doi:10.3389/fpls.2024.1404347)
Supplement: Supplementary file 1 [file DataSheet_1.docx]

**Supporting Information**

| Site (code) | Santurde (S) | Valgañón (V) | Ribavellosa (R) | Santa Marina (M) |
| --- | --- | --- | --- | --- |
| Santurde (S) |  | < 0.001 | 0.003 | 0.062 |
| Valgañón (V) | 0.622 |  | 0.198 | 0.045 |
| Ribavellosa (R) | 0.491 | 0.223 |  | 0.094 |
| Santa Marina (M) | 0.319 | 0.340 | 0.287 |  |

**Table S1.** Correlations (Pearson correlation coefficients) calculated between the site residual series of ring-width indices considering the common period 1988−2022. Correlation coefficients and significance levels are shown in the lower and upper diagonals, respectively.

**Figure S1.** Climate data in the four study sites (V, Valgañón; S, Santurde; R, Ribavellosa; M, Santa Marina). Plots show annual data of: (a) mean maximum (TMx) and (b) minimum temperatures, and (c) total precipitation (Pre). The lowermost plot shows weekly SPEI (24-month long scale, SPEI 24) data. Lines indicate significant (*p* < 0.05) trends according to Kendall tau (τ) tests and different colors or symbols correspond to different sites.

**Figure S2.** Relationships between tree age and mean growth rate (tree-ring width) for each study site (dashed lines) and condidering all trees (thick regression line). Statistics of the linear regressions are shown in the upper right corner.


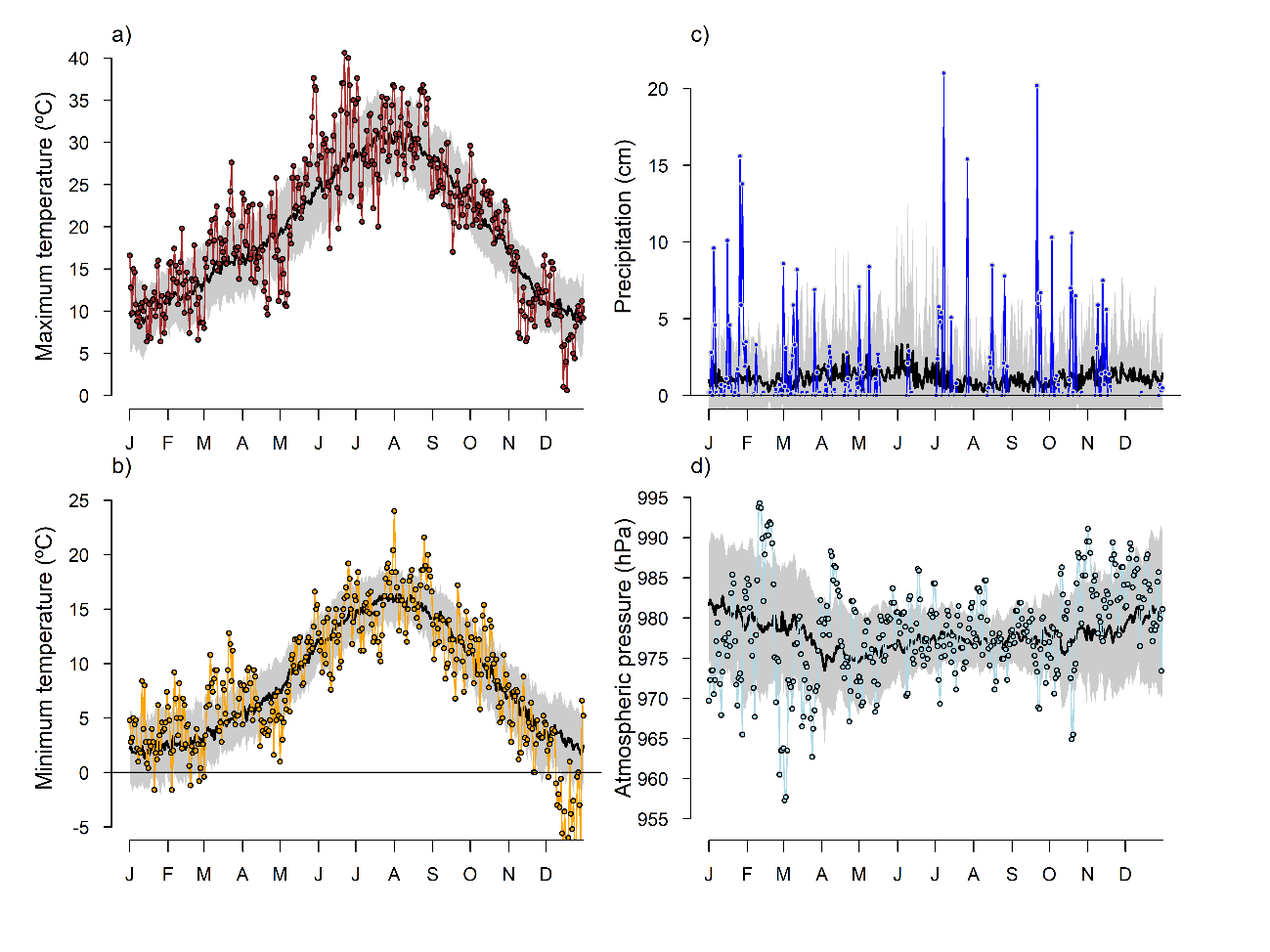


**Figure S3.** The 2001 year in the context of the period 1951−2022. The black line shows the mean value of maximum temperature (a), minimum temperature (b), precipitation (c) and atmospheric pressure (d) according to data from the Logroño airport station. The grey shaded area represents the standard deviation from the mean. The lines and dots represent daily values observed in the year 2001.

| (a) |
| --- |
|  |
| (b) |
|  |

**Figure S4.** The year 2001 was characterized by wet-cool spring conditions followed by dry conditions in early summer. (a) The March 1-month SPEI in the study region was significantly (*p* < 0.05, dashed lines) higher than the 1961−2022 mean indicating exceptional wet and cool conditions in that month. (b) The June 3-month SPEI with the 2001 value (red symbols) was also lower than the 5% confidence interval.
